# Ant nests as a microbial hot spots in a long-term heavy metal-contaminated soils

**DOI:** 10.1007/s11356-021-16384-y

**Published:** 2021-09-16

**Authors:** Beata Klimek, Hanna Poliwka-Modliborek, Irena M. Grześ

**Affiliations:** 1 Institute of Environmental Sciences, Faculty of Biology, Jagiellonian University, Gronostajowa 7, 30-387 Kraków, Poland; 2 Department of Zoology and Animal Welfare, University of Agriculture in Krakow, Mickiewicza 21, 31-120 Kraków, Poland

**Keywords:** Biolog^®^ ECO plates, Community-level physiological profiles (CLLP), *Lasius niger*, Metals, Soil microbial respiration, Metal toxicity index

## Abstract

Interactions between soil fauna and soil microorganisms are not fully recognized, especially in extreme environments, such as long-term metal-polluted soils. The purpose of the study was to assess how the presence of *Lasius niger* ants affected soil microbial characteristics in a long-term metal-polluted area (Upper Silesia in Poland). Paired soil samples were taken from bulk soil and from ant nests and analysed for a range of soil physicochemical properties, including metal content (zinc, cadmium, and lead). Microbial analysis included soil microbial activity (soil respiration rate), microbial biomass (substrate-induced respiration rate), and bacteria catabolic properties (Biolog^®^ ECO plates). Soil collected from ant nests was drier and was characterized by a lower content of organic matter, carbon and nitrogen contents, and also lower metal content than bulk soil. Soil microbial respiration rate was positively related to soil pH (*p* = 0.01) and negatively to water-soluble metal content, integrated into *TI*_ws_ index (*p* = 0.01). Soil microbial biomass was negatively related to *TI*_ws_ index (*p* = 0.04). Neither soil microbial activity and biomass nor bacteria catabolic activity and diversity indices differed between bulk soil and ant nests. Taken together, ant activity reduced soil contamination by metals in a microscale which support microbial community activity and biomass but did not affect Biolog® culturable bacteria.

**Supplementary Information:**

The online version contains supplementary material available at 10.1007/s11356-021-16384-y.

## Introduction

Soil microorganisms are crucial component of terrestrial ecosystems; their activity, biomass, and community structure affect virtually all soil biological processes. Bacteria and fungi are the most important components of soil microbial community (van der Heijden et al. 2008). Besides microbial communities, soil faunas which are animals of different sizes and life history traits are yet another important biological component of soil. Soil fauna facilitates the microbial decomposition of soil organic matter (SOM), both directly by the consumption and fragmentation of SOM and indirectly by their influence on soil microorganisms and vegetation (Schädler and Bradl 2005). Interactions between soil microorganisms and soil fauna are not fully recognized, especially in extreme environments, such as long-term metal-contaminated soils.

Ants are common in metal-polluted areas (e.g. Petal 1978; Nahmani and Lavelle 2002; Migliorini et al. 2004). Although metal pollution may reduce ant colony size and survival, ants are known to be relatively resistant to metal pollution in comparison with other groups of epigeic fauna due to their ability to regulate their internal metal contents (Grześ 2009; Grześ et al. 2019). Ants are “soil engineers”, that is, edaphic organisms that are able to modulate the resources to other organisms through their mechanical activities (Folgarait 1998; Cammeraat and Risch 2008; Farji-Brener and Werenkraut 2017; Viles et al. 2021).

It has been shown that metals reduce microbial activity and biomass (Azarbad et al. 2013) and alter microbial community structure (Azarbad et al. 2015) as well as catabolic and genetic diversity of soil microbial communities (Gołębiewski et al. 2014; Klimek et al. 2016; Pająk et al. 2016; Kuźniar et al. 2018). On the other hand, a lack of effect or even a positive effect of metals on soil microorganisms was also reported (Chodak et al. 2013). Soil microbial community is characterized by a high functional redundancy (Walker 1992), meaning that particular microbial function in soil can be performed by a range of species. However, some microbial groups performing key functions in soil, like nitrifying bacteria, can be metal-sensitive (Bisset et al. 2013). Thus, any negative change in microbial community composition may lead to disruption of specific soil processes.

We aimed to check if the presence of *Lasius niger* ants may be beneficial for soil microorganisms in a long-term metal-polluted region. Krzysztofiak (1991) found that ants reduce the concentrations of heavy metals in their nests comparing surrounding (bulk) soil. Therefore, ant nests in metal-contaminated areas may serve as a microbial hot spots because of lower metal pollution. Since bacteria are known to be more sensitive to metals than fungi (Rajapaksha et al. 2004), in this study, we focused mostly on bacterial part of soil microbial community. We expected that ant nests will be characterized by a higher soil microbial activity and biomass as well as by more functionally diverse bacterial communities in comparison with bulk soil.

## Materials and methods

### Study area

Upper Silesia in Poland is often referred to as an area of ecological disaster (Pawlowski 1990). Exploitation of metal ore deposits in the region is dated since the Middle Ages, and a large-scale industry started in the 1960s. Metal ore deposits and intensive mining and smelting have resulted in high metal contents, mainly zinc, lead, cadmium, and thallium in environment and living organisms, exceeding many times concentrations allowable by law regulations in Poland (i.e. Rozporządzenie Ministra Środowiska 2002; Sowa and Skalski 2019; Magiera et al. 2021). The climate in the Upper Silesia (southern Poland) is temperate, with ca. 700 mm mean annual precipitation and 8 °C mean annual temperature.

Soils were collected in two long-term metal-contaminated regions of Upper Silesia, in vicinity of Olkusz (OLK) and nearby Miasteczko Śląskie (MS), characterized by a different level of metal pollution. Soils of MS region are generally less metal-polluted than OLK region (Rola and Osyczka 2018), as metal ores in the MS region are currently mostly depleted, and only metal processing is taking place there. The most contaminated stands were located near these two metal smelters. and the following locations were typed along increasing distance from the smelters towards north-west. In both OLK and MS transects, seven stands were set, giving altogether 14 studied sites (locations). Transect length in OLK was about 30 km and in MS about 22 km, according to meadow distribution in these region (see Suplementary Material 1).

### Collection of soil samples

On each location, a plot of 400 m^2^ was established, and three *Lasius niger* ant nests were identified, which were typically unevenly distributed. For the plot, if more nests were found, the nests which had larger distances from each other were chosen, keeping a minimal distance between nests on a 5 m. Soil samples were collected using a spade from three nests up to 10 cm depth and mixed to obtain one soil sample per site directly in a plastic box (ca 1 dm^3^ of soil volume). A sample of bulk soil was collected also, keeping a minimal distance from each nest of 2 m. Bulk soil was also composed of three soil subsamples per site and mixed in a separate plastic box (ca 1 dm^3^ of soil volume). In total, 28 mixed soil samples were collected: 2 transects × 7 locations × 2 soil types (ant nests and bulk soil). A mixed soil sample was collected to provide the representativeness of each plot, as many soil properties are characterized by a high spatial variability. Soils were collected in November (2014), which is a low season of ant activity and had limited destructiveness for the ants. Single ant individuals found in some soil samples were removed manually.

### Soil physical–chemical analysis

The dry weight (DW) of the soil samples was determined by measuring the mass loss (water) after soil samples drying at 105 °C for 24 h. Next, the organic matter content (OM) in soil dry weight was determined as the mass loss on ignition at 550 °C for 24 h. The water holding capacity (WHC), which was the amount of water that a given soil can hold without leaking, was measured by a standard gravimetric method after soil soaking for 24 h in net-ended plastic pipes immersed in water. The organic carbon (C), total nitrogen (N), and total sulphur (S) were analysed by dry combustion of ca. 15 mg milled soil samples with an elemental analyser (Vario El III, Elementar Analysensysteme GmbH), and the C:N ratios were calculated for each soil sample. The soil pH was measured in air-dried subsamples (2 g) shaken in deionized water (1:10 w:v) for 1 h at 200 rpm.

The concentrations of the elements, both total and water extractable, were measured using atomic absorption spectrometry (AAS) with a flame or graphite furnace nebulizer, depending on metal concentration (PerkinElmer). The total element concentrations, that is, cations calcium (Ca), potassium (K), magnesium (Mg), and sodium (Na) as well as heavy metals including cadmium (Cd), lead (Pb), and zinc (Zn) in each soil sample, were determined after wet digestion of 0.5 g of DW in 10 ml of SupraPure-concentrated HNO_3_ and HClO_4_ (7:1 v/v) (Sigma-Aldrich). The accuracy of the mineralization process was determined using blank samples as well as standard certified material (CRM025-050, Sandy Loam 8, RT Corp.). Water-soluble metal content in soil was measured after extraction of equivalent of 3 g of DW in 30 ml of deionized water for 0.5 h at 200 rpm. In order to stabilize the solution before analysis, a drop of concentrated HNO_3_ was added into each sample. Each analysis was performed in three subsamples from each soil sample, and the data were averaged and expressed based on the dry weight of the soil.

Study sites are polluted with several metals, mainly Cd, Zn, and Pb (Grześ 2009; Zakrzewska and Klimek 2018); each one is characterized with a different toxicity to living organisms. In order to easy compare metal pollution and toxicity between stands, the soil metal contents on each plot was integrated and expressed by a toxicity index (TI) as follows:

1 $$ TI=\sum \limits_{i=1}^M\left(\frac{C_i}{EC{50}_i}\right) $$

where *M* means the three studied metals, *C*_*i*_ is the concentration of *i* metal in the soil (mg kg^−1^ DW), and *EC50*_*i*_ is the dose of that metal causing a 50% reduction in dehydrogenase activity, an essential endocellular enzyme involved in oxidoreduction processes, a suitable indirect indicator of soil microbial activity (original EC50s taken from Welp 1999). TI indexed were calculated separately for total (*TI*_*t*ot_) and water-soluble metal (*TI*_ws_) concentrations in soil.

### Microbial activity and biomass analysis

Soil samples (equivalents of 50 g DW) were placed in glass vials, adjusted to 60% of their maximum water holding capacity (WHC) and acclimated at 22 °C (±0.5 °C) for 5 days before respiration rate measurements. The samples’ moisture was adjusted daily with deionized water. The soil sample respiration rate was measured by CO_2_ trapping in such a way that each soil sample was placed in an airtight jar with a beaker of 5 ml 0.2 M NaOH (ref). Closed jars were incubated for 1 day (the incubation time was recorded to the nearest minute). After incubation, jars were opened, and 2 ml BaCl_2_ was added to the NaOH solution; the excess sodium hydroxide was titrated using a digital Jencons burette with 0.1 M HCl (0.01 ml precision) in the presence of phenolphthalein as a solution pH (colour) indicator. Several empty jars (with only NaOH) were placed among the other samples as blanks. The soil respiration rate was expressed as mM CO_2_ kg SOM^−1^ 24 h^−1^. The respiration rate was measured twice for each sample and results were averaged.

Subsequently, in these same soil samples, microbial biomass was measured using substrate-induced respiration (SIR) after the addition of glucose solution. Soil samples were amended with glucose solution corresponding to the glucose dose of 10 mg g^−1^ DW soil, which was a concentration causing maximal potential respiration on glucose (data not shown). Water addition with glucose solution did not exceed soil moisture level above 60% WHC. After the glucose solution addition, soil samples were mixed, immediately closed in jars and incubated for 4 h at 22 °C, and soil respiration rate was measured as above. The microbial biomass (SIR-biomass) was calculated from the SIR according to the equation given by Anderson and Domsch (1978), *C*_mic_ (mg g^−1^ OM) = 40.04 *y* + 0.37, where *y* was ml CO_2_ h^−1^ OM g^−1^.

### Biolog^®^ analysis

The catabolic activity and functional diversity of the soil bacteria were analysed using Biolog® ECO plates containing three sets of 31 carbon substrates and tetrazolium dye as the substrate utilization indicator (http://www.biolog.com). The substrates were classified into six substrate guilds, namely, amines, amino acids, carbohydrates, carboxylic acids, polymers, and miscellaneous, according to Dobranic and Zak (1999). Prior to the Biolog^®^ analysis, the field-moist soil samples were wetted up to 60% of their maximal water holding capacity and pre-incubated during 1 week at 22 °C. Subsequently, the equivalent of 3 g of DW soil was shaken for 1 h in 30 ml of a 0.9% NaCl solution (pH 7) and settled for approximately 30 min to decant the soil particles. The suspensions were diluted (10^–2^) in NaCl solution and inoculated onto Biolog® ECO plates (125 μl per well) using a multichannel pipette. All the plates were incubated in the dark at 22 °C, and substrate utilization was measured as the light absorbance at 590 nm (μQuant spectrometer; BIO-TEK Instruments). The first measurement was made immediately after inoculation, and subsequent readings were obtained at 24-h intervals for 168 h. The absorbance measurements for individual wells were corrected against the control well containing only the microbial solution. Absorbance values below 0.06 (spectrometer detection limit) were considered as 0. Each soil sample was analysed in three replicates (one Biolog^®^ ECO plate per one soil sample); the data were averaged.

The general bacterial activity was calculated as the area under the curve (AUC) using the following formula:

2 $$ AUC=\sum \limits_{i=1}^N\sum \limits_{t=1}^{n-1}{A}_n+{A}_{\mathrm{n}+1}2\times \left({t}_{n+1}-{t}_n\right) $$

where *A*_n_ and *A*_n+1_ are the absorbance of each individual well at two consecutive measurements at times *t*_n_ and *t*_n+1_; *n* represents particular measurements (scorings); and *N* represents the number of substrates on the plate (31 for ECO plates).

Because both the density and the activity of microbial cells affect the rate of colour development, the functional diversity index (*H*’ and *R*) and community-level physiological profiles (CLPP) were compared on the same sample average well colour development (AWCD), calculated as the mean well absorbance. For further calculations, catabolic profile at AWCD value of 0.15 was used, irrespective of the incubation time (Preston-Mafham et al. 2002). The absorbance values for individual wells/substrates were expressed as a proportion of the total sample absorbance on the plate and standardized to 1 for each sample.

The number of substrates used by the bacteria (*R*, substrate richness) was counted for each sample. The bacterial functional diversity index *H*’, derived from the Shannon–Wiener biodiversity index, which is based on the structure of substrate use:

3 $$ H^{\prime }=-{\sum}_{i=1}^s{p}_s\left({\mathit{\log}}_{10}{p}_s\right) $$

which was derived from the number of substrate *s* decayed by bacteria on a set of 31 substrates and the utilization of an individual substrate *p*_s_ calculated as the absorbance for a given well divided by the sum of absorbance for all wells.

### Statistical analysis

In order to verify the possible differences between bulk and ant nest soils in terms of physical and chemical characteristics, both soils were compared using paired *t* test for groups with unequal variance (as the data were characterized by a high variability). The differences were consider to be significant when *p* < 0.05.

The second step of analysis was conducted to detect which of the environmental factors affected microbial parameters. Soil respiration rate, microbial biomass, AUC, *H*’, and *R* were analysed separately using general linear models (GLM) with categorical variables: soil type (ant nests or bulk soil) and transect (OLK or MS) and with quantitative (linear) variables, *TI*_ws_ index as a combined measure of soil metal pollution, soil pH as an important soil trait affecting metal mobility, and exclusively for AUC, *H*’, and *R* also soil C content (%) as a representation of soil organic matter amount, a factor fostering for metal binding in soil. The data distribution was checked and they were transformed if needed. The differences were consider to be significant when *t* < 0.05.

Non-parametric multidimensional scaling analysis (NMDS) with the Bray–Curtis similarity measure was used to compare bacterial CLPP profiles for bulk soil and ant nests substrate. This analysis was performed first separately for two transects and then, because of their similarity, jointly for both transects. A stress value lower than 0.2 is considered to be a good representation by NMDS maps of the set of information included in the rank of the similarity matrix.

*t* test and GLM analyses were conducted using Statgraphics Centurion 18 software (Stat-Point Technologies Inc., Warrenton, VA, USA) and multivariate analyses using PAST 2.17c software (Natural History Museum, University of Oslo, Norway).

## Results

### Soil physical and chemical properties in ant nest soil and bulk soil

Soil samples collected from ant nests were drier than bulk soil, meaning field moisture (87.2% and 79.9%, respectively; *p* = 0.01), and were characterized by a lower content of organic matter (3.4% and 6.4%, respectively; *p* = 0.01) (Table 1). Soil samples collected were characterized by a generally low content of soil organic matter with a mean value of 4.9% for all samples, which was reflected in a low value of water holding capacity (mean of 54.7%) and also in low contents of essential elements, that is, C, N, and S (3.05%, 0.22%, and 0.03%, respectively). Soil samples collected from ant nests contained nearly less than half of carbon than bulk soil (38% less; *p* = 0.04), nitrogen (38% less; *p* = 0.05), and sulphur (53% less; *p* = 0.04) (Table 1). In turn, water holding capacity (WHC) and basic cation content (Ca, Mg, K, Na) did not differ between ant nests and bulk soil (Table 1), which could be resulted from high variability in these parameters observed. The studied soils were characterized by pH close to the neutral with mean value for all samples of 6.55 and did not differ between ant nests soil and bulk soil (*p* = 0.34) (Table 1).

**Table 1:** Mean values (underlined), standard deviations, and minimal and maximal values for physical and chemical properties of the studied soils: dry mass content (DW), organic matter content (OM), water holding capacity (WHC), soil pH, contents of carbon (C), nitrogen (N), sulphur (S), carbon to nitrogen ratio (C:N), contents of calcium (Ca), potassium (K), magnesium (Mg), and sodium (Na); total contents of cadmium (Cd_tot_), lead (Pb_tot_), and zinc (Zn_tot_); water-soluble contents of cadmium (Cd_ws_), lead (Pb_ws_), and zinc (Zn_ws_); and toxicity indexes calculated based on total (*TI*_tot_) and water-soluble metal concentrations (*TI*_ws_). Significant differences between soils in paired *t* test (*p* < 0.05) are bolded

Soil property	Unit		Data set values							
		*p* value	Bulk soil				Ant nests			
			Mean	SD	Min	Max	Mean	SD	Min	Max
DW	% W	**0.01**	79.9	8.4	58.0	89.1	87.2	4.0	79.0	92.8
OM	% DW	**0.01**	6.4	4.6	2.6	20.6	3.4	1.2	2.1	6.0
WHC	% DW	0.22	59.8	24.6	34.6	127.6	49.5	18.0	32.4	91.9
pH	-	0.34	6.39	0.95	4.88	7.89	6.71	0.81	5.65	8.35
C	% DW	**0.04**	3.8	2.3	1.4	9.6	2.3	1.0	1.3	4.4
N	% DW	**0.05**	0.3	0.2	0.1	0.8	0.2	0.1	0.1	0.3
S	% DW	**0.04**	0.04	0.03	0.00	0.12	0.02	0.02	0.00	0.06
C:N	-	0.92	14.4	3.8	11.7	26.7	14.5	5.3	11.0	32.0
Ca	mg kg^−1^ DW	0.57	9322	15230	779	54419	6474	10595	494	31145
K	mg kg^−1^ DW	0.60	1339	897	375	3954	1144	1029	362	4273
Mg	mg kg^−1^ DW	0.25	1869	2347	400	7545	1054	994	248	3311
Na	mg kg^−1^ DW	0.57	101	71	45	317	86	60	3	224
Cd_tot_	mg kg^−1^ DW	0.08	7.4	6.6	0.3	18.5	3.6	4.0	0.2	14.6
Pb_tot_	mg kg^−1^ DW	0.19	514	562	22	1581	275	350	20	1206
Zn_tot_	mg kg^−1^ DW	**0.04**	1383	1585	39	4523	391	453	36	1401
Cd_ws_	mg kg^−1^ DW	**0.01**	0.35	0.36	0.01	0.97	0.03	0.03	0.00	0.11
Pb_ws_	mg kg^−1^ DW	**0.01**	17.7	19.0	0.6	56.5	1.7	2.5	0.0	7.3
Zn_ws_	mg kg^−1^ DW	**0.02**	49.8	60.1	2.0	166.9	4.5	5.1	0.5	16.3
*TI*_tot_	-	**0.04**	12.9	14.3	0.4	40.3	3.9	4.3	0.3	13.1
*TI*_ws_	-	**0.00**	708	704	27	2004	67	84	4	248

Soils on some locations were characterized by a high metal pollution, ranging as high as 4523 mg kg^−1^ DW for total Zn, 1581 mg kg^−1^ DW for total Pb, and 18.5 mg kg^−1^ DW for total Cd content, whereas water-soluble metal concentrations corresponded to a few percent of total metal concentrations (Table 1). Total zinc content was found to be significantly lower in ant nests than in bulk soil (72% less; *p* = 0.04) as well as water-soluble zinc contents (91% less; *p* = 0.02), cadmium (92% less; *p* = 0.01), and lead (90% less; *p* = 0.01) (Table 1). In turn, total content of cadmium and lead did not differ between soils, presumably because of high variability in these data (Table 1).

For both *TI*_tot_ and *TI*_ws_ index values, the most influential metal was zinc, which is a dominating pollutant in the region. *TI*_tot_ was lower on the order of magnitude in ant nests soil in comparison to bulk soil (3.86 and 12.89, respectively; *p* = 0.04). *TI*_ws_ was also as much lower in ant nests than in bulk soil (67 and 708, respectively; *p*<0.001) (Table 1).

### Soil microbiological properties in ant nest soil and bulk soil

None of the measured soil microbial properties differed between bulk soil and ant nests (Table 2). Soil microbial respiration rate in individual samples ranged from 9.20 to 38.5 mM CO_2_ OM kg^−1^ 24 h^−1^. GLM analysis indicated that soil microbial respiration of bulk soil and ant nests did not differ between each other; also effects from transect were not significant (Table 3). We found, however, that microbial respiration rate was positively related to soil pH (*p* = 0.01) and negatively to *TI*_ws_ (*p* = 0.01) (*R*^2^_adj_=25.6%; model *p* < 0.01).

**Table 2:** Soil microbial properties of the studied soils. RESP denotes soil respiration rate, SIR-biomass denotes soil microbial biomass, AUC denotes soil bacteria activity, and *H*’ and *R* represent measures of soil bacteria functional diversity. None of the microbial parameters differed between bulk soil and ant nests or between transects

Transect	Distance from the smelter (~km)	RESP (mM CO_2_ OM kg^−1^ 24 h^−1^)		SIR-biomass (mg OM g^−1^)		AUC		*H*’		*R*	
		Bulk soil	Ant nests	Bulk soil	Ant nests	Bulk soil	Ant nests	Bulk soil	Ant nests	Bulk soil	Ant nests
OLK	1	23.79	26.80	0.89	1.00	28.74	19.69	0.96	1.00	17.0	24.3
	1	20.09	30.70	0.75	1.15	31.12	56.32	1.06	1.15	21.7	27.0
	2	9.94	33.98	0.37	1.27	16.21	65.59	0.91	1.10	19.3	21.3
	5	9.20	19.94	0.34	0.75	52.70	35.37	1.19	1.18	27.0	28.0
	10	21.86	33.99	0.82	1.27	32.77	61.48	1.07	1.24	25.7	26.3
	20	16.48	35.23	0.62	1.32	43.17	38.69	1.24	1.13	28.0	25.0
	30	26.75	34.47	1.00	1.29	51.24	26.87	1.14	1.02	24.3	21.3
MS	2	17.87	33.28	0.67	1.24	52.18	47.01	1.14	1.16	26.7	27.3
	2	31.30	21.00	1.17	0.79	35.62	16.12	0.94	1.09	23.3	28.7
	4	12.00	13.02	0.45	0.49	44.87	46.90	1.06	1.11	24.0	23.7
	7	26.41	23.65	0.99	0.88	87.80	59.52	1.17	1.08	25.3	22.3
	15	32.41	30.72	1.21	1.15	6.78	48.52	1.17	1.06	25.0	21.0
	17	19.08	14.77	0.71	0.55	14.18	6.71	1.06	1.11	20.3	20.3
	22	35.19	38.50	1.32	1.44	21.22	39.72	0.84	1.04	18.7	25.7

odel *p* = 0.004).

**Table 3:** Results of the GLM analysis (*p* values) for soil respiration rate (RESP), microbial biomass (SIR-biomass), soil bacteria activity (AUC), and soil bacteria functional diversity (*H*’, *R*) with categorical variables: soil type (ant nests or bulk soil) and transect (OLK or MS) and with quantitative (linear) variables: *TI*_ws_ index, soil pH, and (exclusively for AUC, *H*’, and *R*) soil C content (%) (*n* = 28). Sign “x” denotes that the factor (soil C content) was not included in the model for RESP and SIR-biomass; sign “-” denotes that the factor was not significant and was removed from a model

	RESP	SIR-biomass	AUC	*H*’	*R*
Model (*R*^2^_adj_)	0.006 (28.1%)	0.004 (24.3%)	0.339 (0.0%)	0.312 (0.3%)	0.324 (0.1%)
Soil type	-	-	-	0.312	0.324
Transect	-	-	-	-	-
*TI*_ws_	0.020	0.004	-	-	-
pH	0.017	-	-	-	-
C (%)	x	x	-	-	-

Soil microbial biomass values ranged from 0.34 to 1.44 mg OM g^−1^ (Table 3). The only significant factor in GLM for soil microbial biomass was *TI*_ws_ which affected microbial biomass negatively (*p* = 0.004) (*R*^2^_adj_=24.3%; m

GLM models for bacterial indices appeared to be not significant, showing lack of effects from soil type, transect, metal concentration, soil pH and soil C content. Bacteria activity and catabolic diversity indices calculated based on Biolog^®^ plate absorbance values were relatively high. More than a half of carbon compounds (from set of 31) were used by bacteria for each soil sample (Table 3). Detailed data for each sample can be found in Electronic Supplementary Material. Carboxylic acids were the most intensively used group of substrates on plates (Supplementary Material 3).

**Fig. 1:**
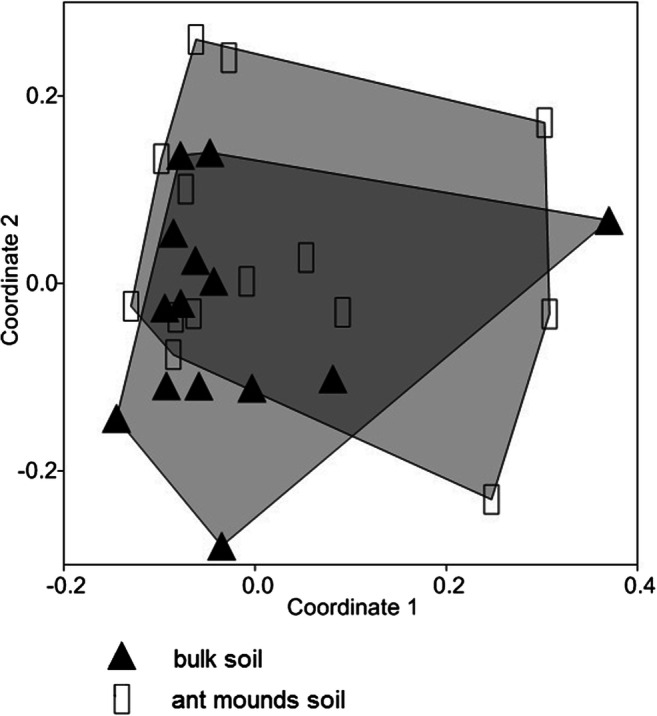
Plot of the two dimensions of NMDS analysis of CLPP profiles between bulk soil and ant mounds soil

CLPP pattern did not differ between ant nest soil and bulk soil. CLPP profiles between bulk soils and ant nests were strongly overlapping as showed in Fig. 1. Two NMDS dimensions allowed to obtain a stress value of 0.169.

## Discussion

Soil pollution with heavy metal is a serious problem in some regions of the world, for example, as a result of massive metal processing, as in the Upper Silesia region. The concentrations of metals measured in a current study were similar to these observed in earlier studies carried (Klimek 2012; Azarbad et al. 2013; Pająk et al. 2016; Gruszecka-Kosowska and Kicińska 2017). As soils can be contaminated with a mixture of metals characterized by a diverse toxicity to soil organisms, assessment overall metal pollution can be performed using toxicity index (Welp 1999). In our study, TI for total metal concentrations ranged from 0.3 to 40.3 and for water-soluble metal concentrations ranged from 4 to 2004, being comparable with other studies conducted in the region (Azarbad et al. 2015). By comparison, toxicity index for total metal concentrations as high as 250 was found near Avonmouth smelter in the UK (Stefanowicz et al. 2008). Standarization of metal pollution level allows also to compare metal effect on soil organisms between different stands and also between different environments within a stand, even because metals differ in their mobility in soils. In a current study, we found that metal toxicity index decreased roughly with distance from the smelter and also differ between soil in *Lasius niger* nests and bulk soil. Toxicity indexes calculated for both total and water-soluble metal concentrations indicated that soil in ant nests was less contaminated than bulk soil.

We observed also that other than metal soil physical and chemical properties differed between bulk soil and *Lasius niger* nests. For instance, nest soil was drier than bulk soil and contains less organic matter. Such differences in soil physical–chemical properties between bulk soil and ant nests can be attributed to ant activity, i.e. food storage and accumulation of faeces and ant remains. Ants may change soil structure in different ways, i.e. though increased soil porosity and aeration or reduced bulk density (Dostál et al. 2005; Bierbaß et al. 2015). Altered soil structure in ant nests may therefore results in increasing soil leaching by rainfall and metal eluviation down to the soil profile.

Despite studied ant species affected soil physical–chemical properties, it was not reflected in soil microbiological features. We did not observe differences between ant nests and bulk soil in microbial respiration rate and biomass nor in soil bacteria activity and catabolic diversity indices measured using Biolog® plates. Such lack of effect could be driven by a several reasons. First, we conducted the study during low season of ant activity; therefore, their effect on soil microorganisms could not be as expressive as during vegetation season. Second, the ant effect on soil microorganisms may depend on its species. We used omnivorous *Lasius niger* because of its common occurrence in metal-polluted regions. However, this species may not affect substantially soil microorganisms as much as other ant species (Wagner et al. 1997; Farji-Brener and Werenkraut 2017). Jílková et al. (2017) who studied three ant species in metal-polluted region in Czech Republic found that *Lasius niger* nests did not differ from the surrounding soil in soil microbial respiration rate and microbial biomass. However, the authors assumed that this could be caused by relatively high soil pH in their study. In fact, similar soil conditions were found in our study, that is, high metal pollution and relatively high soil pH. Soils in our study were mostly neutral towards alkaline, which result from bedrock properties in the region being a part of the Polish Jurassic Highland — a geological formation composed of Mesozoic limestones and dolomites. Higher soil pH fosters the metal binding; we found that water-soluble metal fraction does not exceed few percent of total metal concentrations.

The third explanation may be related to the fact that ants may produce antifungal and antibacterial agents that are secreted by the paired thoracic metapleural gland which may suppress microbial activity in nest (Beattie 2010). Some of the enzymes produced by bacterial strains living in *Lasius niger* gut can possibly affect free-living soil organisms (Díez-Méndez et al. 2019). Last but not least, these could be a result from applied method limitations, especially Biolog^®^ plates which represents only a culturable fraction of soil microorganisms. Amador and Görres (2007) also did not found ant effect on soil bacteria using Biolog® ECO plates. Another possible explanation for a lack of difference between ant nests and bulk soil could be from factors not tested in this study, for example, effects of pollutants other than metals, for example, total petroleum hydrocarbons (TPH), which affect soil microorganisms differently than metals do (see Klimek et al. 2016).

We did not find a difference in soil pH between bulk soil and ant nests. Reports about ant effect on soil pH are inconsistent; some studies have shown an increase of pH in ant nest (Dostál et al. 2005), while other reported a decrease (Amador and Görres 2007) or a lack of effect (Leal et al. 2007). The latest meta-analysis study of Farji-Brener and Werenkraut (2017) showed that ant nest have no overall effect on soil pH, and the positive/negative effects appear only in some environmental conditions. Dauber and Wolters (2000) and Jílková et al. (2017) who studied *Lasius niger* between other ant species also did not find such difference. In turn, Bierbaß et al. (2015) found that soil pH was higher in ant nest than in bulk soil; however, they studied other *Lasius* species, namely, *Lasius flavus*. Ant effect on soil pH may highly depends on its species, in particular on ant regiment and food preferences and the surrounding soil (environment) properties. Cammeraat and Risch (2008) suggested that ants regulate soil pH in alkaline than in neutral or acidic soils, and these could be due to faster carbon turnover in nests. Fast carbon turnover in nests leads to the production of organic acids, which then leads to a decrease in pH. We did not observe such effect in our study. Ant effect on soil pH might be lesser in metal-polluted soils than in unpolluted ones, as colonies tend to be smaller. Jílková et al. (2017) in a similar study with *Lasius niger* in metal-polluted region explained such failure that the nests tested could be too young to have significantly affected substrate properties.

Nest microbial communities are assumed to reflect the behaviour of the hosting ant species (Amador and Görres 2007; Boots et al. 2021). *Lasius niger* used to collect nectar from flowering plants (Gorb and Gorb 2019) and honeydew produced by aphids (Offenberg 2001); therefore, we expected that bacteria from ant nests will prefer carbohydrates use on Biolog^®^ plates compared to bacteria from bulk soil. However, returning to issue of a low season of ant activity meaning that ants were not active — this was addressed also to plants, aphids, or soil microorganisms. On the other hand, the studied soils were characterized by a low content of organic matter (4.9% on average) which was reflected in low soil water holding capacity (55% on average); both these factors limit soil microbiological activity in general (Romero-Freire et al. 2016).

In our study, we focused on bacteria, because they are known to be more sensitive to environmental factors including metal pollution than fungi, as they have higher surface area-to-volume ratio than fungi, and bacteria usually do not have cell walls (Ledin 2000). However, ant activity may affect rather the fungal part of the community. Fungi are thought to correspond majority of soil microbiological activity, including soil respiration rate (Strickland and Rousk 2010). In a 2-year laboratory experiment, Brinker et al. (2019) showed that differentiation in bacterial community composition between ant nest and surrounding soil varied over the time, whereas fungal communities in the nest are actively managed by ants. Interactions of ants with pathogenic fungal species are relatively well studied, but little is known about interactions with free-living soil fungi (Malagocka et al. 2019), especially in metal-polluted soils, which remains promising space for further research.

We found a negative metal effect on soil microbial activity and biomass but a lack of metal effect on culturable soil bacteria, including CLPP profiles. These may suggest that in the studied soils with a long-term metal contamination, bacteria developed tolerance to metals (Piotrowska-Seget et al. 2005). We found that soil microbial respiration rate was positively related to soil pH. Soil pH is known as a crucial factor regulating soil microbial activity through the effect on carbon substrate availability for microorganisms and effects on microbial diversity (Lauber et al. 2009). However, the positive effect of soil pH on microbial communities is most evident in a lower (suboptimal) soil pH range, up to 5.0, and above this value seems to be less significant (Kaiser et al. 2016).

Lower metal content in ant nests in comparison with the surrounding soil in long-term metal-polluted region has no direct outcome in enhancing soil microbial properties. Bierbaß et al. (2015) suggested that ant nests represent rather cold spots not hot spots for C cycling comparing to surrounding (bulk) soil. They indicate that lower amount of soil organic carbon in ant nests is the major reason for the diminished carbon mineralization rate, which is in line with our results. Nevertheless, ant activity resulted in increasing soil spatial heterogeneity may be beneficial for other soil organisms.

## Conclusions

Soil collected from *Lasius niger* nests differed from surrounding soil in many physical–chemical properties, including lower metal content. Neither soil microbial activity and biomass nor bacteria catabolic activity or diversity indices differed between bulk soil and ant nests. Taken together, ant activity reduced soil contamination by metals in a microscale, but this was not reflected in soil bacteria activity and catabolic diversity.

## Supplementary information

ESM 1 (DOCX 41 kb)

## Data Availability

The datasets generated during the current study are available in the Electronic Supplementary Material.
