# Supplementary material for: Ant nests as a microbial hot spots in a long-term heavy metal-contaminated soils
Source: Environ Sci Pollut Res Int. 2021 Sep 16;29(7):10848–57. doi: 10.1007/s11356-021-16384-y (PMC8783854; doi:10.1007/s11356-021-16384-y)
Supplement: Supplementary file 1 — (DOCX 41 kb) [file 11356_2021_16384_MOESM1_ESM.docx]

Article title: “**Ant nests as a microbial hot-spots in a long-term heavy metal contaminated soils**”

Journal: “Environmental Science and Pollution Research”

Authors: Beata Klimek*, Hanna Poliwka, Irena Grześ

*- corresponding author, e-mail: [beata.klimek@uj.edu.pl](mailto:beata.klimek@uj.edu.pl)

Electronic Suplementary Material 1: Location of studied stands.

| Sample number  (a pair per site:  odd numbers – bulk soil,  even numbers – ant nests) | transect | GPS location | | Distance  from the smelter (~km) |
| --- | --- | --- | --- | --- |
|  |  | N | E |  |
| 1 – 2 | OLK | 50°17' | 19°28' | 1 |
| 3 – 4 |  | 50°17' | 19°28' | 1 |
| 5 – 6 |  | 50°17' | 19°29' | 2 |
| 7 – 8 |  | 50°18' | 19°30' | 5 |
| 9 – 10 |  | 50°18' | 19°34' | 10 |
| 11 – 12 |  | 50°23' | 19°40' | 20 |
| 13 – 14 |  | 50°27' | 19°41' | 30 |
| 15 – 16 | MS | 50°29' | 18°56' | 2 |
| 17 – 18 |  | 50°30' | 18°56' | 2 |
| 19 – 20 |  | 50°31' | 18°57' | 4 |
| 21 – 22 |  | 50°33' | 18°57' | 7 |
| 23 – 24 |  | 50°36' | 18°57' | 15 |
| 25 – 26 |  | 50°38' | 18°57' | 17 |
| 27 – 28 |  | 50°40' | 18°55' | 22 |

Electronic Suplementary Material 2: Detailed physical-chemical data for collected soils.

| Soil property | Unit | Soil sample number | | | | | | | | | | | | | | | | | | | | | | | | | | |  |
| --- | --- | --- | --- | --- | --- | --- | --- | --- | --- | --- | --- | --- | --- | --- | --- | --- | --- | --- | --- | --- | --- | --- | --- | --- | --- | --- | --- | --- | --- |
|  |  | 1 | 2 | 3 | 4 | 5 | 6 | 7 | 8 | 9 | 10 | 11 | 12 | 13 | 14 | 15 | 16 | 17 | 18 | 19 | 20 | 21 | 22 | 23 | 24 | 26 | 26 | 27 | 28 |
| OM | % DW | 4.3 | 5.2 | 2.9 | 20.6 | 5.5 | 3.3 | 6.8 | 4.2 | 5.8 | 10.8 | 6.7 | 3.4 | 7.4 | 2.6 | 2.8 | 2.4 | 4.4 | 4.7 | 2.6 | 2.1 | 5.3 | 2.4 | 3.4 | 6.0 | 3.9 | 2.4 | 3.6 | 2.5 |
| WHC | % DW | 54.3 | 34.6 | 127.6 | 41.3 | 54.5 | 49.5 | 93.7 | 54.8 | 55.3 | 76.1 | 55.6 | 43.7 | 56.0 | 40.4 | 36.7 | 42.4 | 91.9 | 62.2 | 39.6 | 32.4 | 81.8 | 36.1 | 43.1 | 59.5 | 38.7 | 40.3 | 49.1 | 39.3 |
| pH | - | 7.3 | 6.4 | 6.7 | 5.3 | 7.6 | 6.6 | 7.9 | 6.2 | 6.4 | 5.2 | 7.3 | 5.5 | 4.9 | 6.0 | 7.4 | 6.3 | 6.2 | 5.9 | 7.9 | 6.7 | 8.4 | 6.9 | 6.4 | 5.7 | 7.7 | 6.4 | 5.9 | 6.6 |
| C | % DW | 2.8 | 2.8 | 1.4 | 9.6 | 3.9 | 2.1 | 3.6 | 3.2 | 3.6 | 6.4 | 6.1 | 2.0 | 3.8 | 1.5 | 1.7 | 1.5 | 2.5 | 3.5 | 2.1 | 1.3 | 3.0 | 1.9 | 2.2 | 4.4 | 3.8 | 1.5 | 2.0 | 1.4 |
| N | % DW | 0.2 | 0.2 | 0.1 | 0.8 | 0.3 | 0.2 | 0.3 | 0.2 | 0.3 | 0.5 | 0.2 | 0.2 | 0.3 | 0.1 | 0.1 | 0.1 | 0.2 | 0.3 | 0.1 | 0.1 | 0.2 | 0.1 | 0.2 | 0.3 | 0.1 | 0.1 | 0.2 | 0.1 |
| S | % DW | 0.04 | 0.06 | 0.00 | 0.10 | 0.03 | 0.02 | 0.02 | 0.05 | 0.05 | 0.06 | 0.12 | 0.01 | 0.03 | 0.01 | 0.02 | 0.01 | 0.02 | 0.03 | 0.02 | 0.01 | 0.05 | 0.01 | 0.02 | 0.03 | 0.06 | 0.00 | 0.01 | 0.00 |
| C:N | - | 16.0 | 12.0 | 13.0 | 13.0 | 14.3 | 11.7 | 13.7 | 15.3 | 14.3 | 13.0 | 26.7 | 13.0 | 11.7 | 13.7 | 14.0 | 11.0 | 12.7 | 13.0 | 16.7 | 11.0 | 13.0 | 15.7 | 14.3 | 13.0 | 32.0 | 12.3 | 12.0 | 13.0 |
| Ca | % DW | 15196 | 2210 | 1473 | 2272 | 22043 | 1446 | 25251 | 1179 | 1904 | 1653 | 53419 | 779 | 899 | 785 | 3880 | 1533 | 2279 | 942 | 21009 | 1054 | 31145 | 494 | 724 | 731 | 24643 | 808 | 692 | 705 |
| K | % DW | 1123 | 1142 | 1277 | 744 | 1020 | 1431 | 3954 | 375 | 2197 | 611 | 1564 | 1007 | 1717 | 583 | 1076 | 670 | 535 | 373 | 864 | 1018 | 4273 | 362 | 1863 | 474 | 730 | 1173 | 1968 | 644 |
| Mg | % DW | 6869 | 1326 | 1353 | 686 | 817 | 701 | 2798 | 400 | 1203 | 489 | 7545 | 545 | 1020 | 416 | 2347 | 568 | 557 | 366 | 771 | 516 | 2772 | 248 | 930 | 341 | 3311 | 601 | 1008 | 416 |
| Na | % DW | 84 | 92 | 88 | 70 | 52 | 87 | 190 | 68 | 89 | 56 | 317 | 73 | 98 | 45 | 115 | 82 | 35 | 41 | 50 | 78 | 189 | 45 | 111 | 3 | 224 | 66 | 98 | 73 |
| Cd_tot_ | mg kg^-1^ DW | 16 | 15 | 11 | 16 | 1 | 2 | 2 | 5 | 18 | 8 | 7 | 1 | 1 | 0 | 10 | 2 | 3 | 5 | 3 | 1 | 1 | 3 | 15 | 4 | 4 | 1 | 0 | 0 |
| Zn_tot_ | mg kg^-1^ DW | 4523 | 3209 | 3725 | 1823 | 215 | 143 | 152 | 722 | 1055 | 546 | 3072 | 64 | 70 | 39 | 1401 | 138 | 179 | 603 | 457 | 113 | 124 | 145 | 628 | 241 | 1310 | 53 | 51 | 36 |
| Pb_tot_ | mg kg^-1^ DW | 519 | 933 | 465 | 1581 | 50 | 59 | 37 | 343 | 1277 | 389 | 1428 | 49 | 51 | 22 | 541 | 98 | 126 | 1206 | 86 | 52 | 20 | 154 | 645 | 227 | 614 | 36 | 26 | 20 |
| Cd_ws_ | mg kg^-1^ DW | 1 | 1 | 1 | 1 | 0 | 0 | 0 | 0 | 1 | 0 | 0 | 0 | 0 | 0 | 0 | 0 | 0 | 0 | 0 | 0 | 0 | 0 | 0 | 0 | 0 | 0 | 0 | 0 |
| Zn_ws_ | mg kg^-1^ DW | 167 | 99 | 162 | 45 | 8 | 6 | 5 | 21 | 44 | 19 | 112 | 3 | 3 | 2 | 16 | 2 | 7 | 3 | 10 | 2 | 2 | 1 | 4 | 1 | 13 | 1 | 1 | 1 |
| Pb_ws_ | mg kg^-1^ DW | 21 | 28 | 21 | 37 | 1 | 2 | 1 | 12 | 50 | 15 | 56 | 2 | 1 | 1 | 5 | 0 | 4 | 5 | 0 | 0 | 0 | 0 | 1 | 0 | 7 | 0 | 0 | 0 |
| TI_tot_ | - | 40.3 | 29.5 | 33.2 | 18.4 | 2.0 | 1.4 | 1.4 | 6.9 | 11.3 | 5.4 | 29.0 | 0.6 | 0.7 | 0.4 | 13.1 | 1.4 | 1.8 | 7.1 | 4.1 | 1.1 | 1.1 | 1.5 | 6.6 | 2.5 | 12.4 | 0.5 | 0.5 | 0.3 |
| TI_ws_ | - | 0.14 | 0.11 | 0.15 | 0.09 | 0.11 | 0.11 | 0.14 | 0.10 | 0.13 | 0.12 | 0.10 | 0.10 | 0.10 | 0.11 | 0.03 | 0.02 | 0.10 | 0.01 | 0.04 | 0.03 | 0.04 | 0.01 | 0.01 | 0.01 | 0.03 | 0.02 | 0.03 | 0.03 |

Electronic Suplementary Material 3: Detailed microbiological data for collected soils.

| Soil property | Unit | Soil sample number | | | | | | | | | | | | | | | | | | | | | | | | | | |  |
| --- | --- | --- | --- | --- | --- | --- | --- | --- | --- | --- | --- | --- | --- | --- | --- | --- | --- | --- | --- | --- | --- | --- | --- | --- | --- | --- | --- | --- | --- |
|  |  | 1 | 2 | 3 | 4 | 5 | 6 | 7 | 8 | 9 | 10 | 11 | 12 | 13 | 14 | 15 | 16 | 17 | 18 | 19 | 20 | 21 | 22 | 23 | 24 | 26 | 26 | 27 | 28 |
| RESP | mM CO2 OM kg^-1^ 24 h^-1^ | 23.8 | 20.1 | 9.9 | 9.2 | 21.9 | 16.5 | 26.8 | 17.9 | 31.3 | 12.0 | 26.4 | 32.4 | 19.1 | 35.2 | 26.8 | 30.7 | 34.0 | 19.9 | 34.0 | 35.2 | 34.5 | 33.3 | 21.0 | 13.0 | 23.6 | 30.7 | 14.8 | 38.5 |
| SIR-biomass | mg OM g^-1^ | 1450.9 | 2066.7 | 928.7 | 666.4 | 1841.9 | 2126.1 | 704.1 | 1404.6 | 1410.1 | 883.0 | 1467.3 | 2484.3 | 1384.8 | 2593.6 | 1360.7 | 2030.6 | 2101.5 | 1153.5 | 2335.6 | 2970.8 | 1870.4 | 2048.2 | 935.5 | 1230.1 | 1389.4 | 2254.2 | 1723.2 | 3249.4 |
| AUC | - | 28.7 | 31.1 | 16.2 | 52.7 | 32.8 | 43.2 | 51.2 | 52.2 | 35.6 | 44.9 | 87.8 | 6.8 | 14.2 | 21.2 | 19.7 | 56.3 | 65.6 | 35.4 | 61.5 | 38.7 | 26.9 | 47.0 | 16.1 | 46.9 | 59.5 | 48.5 | 6.7 | 39.7 |
| H’_bact_ | - | 1.0 | 1.1 | 0.9 | 1.2 | 1.1 | 1.2 | 1.1 | 1.1 | 0.9 | 1.1 | 1.2 | 1.2 | 1.1 | 0.8 | 1.0 | 1.1 | 1.1 | 1.2 | 1.2 | 1.1 | 1.0 | 1.2 | 1.1 | 1.1 | 1.1 | 1.1 | 1.1 | 1.0 |
| R | - | 17.0 | 21.7 | 19.3 | 27.0 | 25.7 | 28.0 | 24.3 | 26.7 | 23.3 | 24.0 | 25.3 | 25.0 | 20.3 | 18.7 | 24.3 | 27.0 | 21.3 | 28.0 | 26.3 | 25.0 | 21.3 | 27.3 | 28.7 | 23.7 | 22.3 | 21.0 | 20.3 | 25.7 |
| amines | % of use | 10.3 | 20.2 | 1.8 | 22.7 | 19.3 | 14.4 | 9.6 | 21.7 | 0.6 | 34.5 | 31.1 | 3.3 | 7.8 | 11.2 | 0.6 | 17.9 | 19.3 | 18.7 | 28.9 | 21.8 | 25.3 | 17.1 | 47.1 | 29.7 | 27.2 | 13.8 | 12.4 | 13.6 |
| amino acids | % of use | 41.5 | 78.5 | 59.5 | 73.9 | 39.4 | 53.8 | 42.1 | 87.6 | 86.1 | 72.8 | 64.9 | 25.3 | 47.4 | 72.8 | 82.6 | 54.9 | 60.9 | 68.6 | 59.8 | 78.9 | 56.1 | 65.5 | 19.2 | 98.8 | 49.6 | 71.2 | 40.9 | 71.0 |
| carbohydrates | % of use | 35.8 | 49.3 | 25.1 | 46.5 | 51.6 | 75.2 | 49.5 | 45.1 | 97.5 | 25.3 | 32.3 | 97.2 | 82.9 | 30.6 | 50.6 | 28.7 | 38.5 | 69.5 | 34.7 | 38.9 | 41.1 | 42.9 | 68.2 | 34.0 | 56.8 | 28.0 | 128.9 | 39.1 |
| carboxylic acids | % of use | 112.0 | 82.1 | 153.5 | 65.8 | 56.4 | 75.1 | 78.9 | 43.9 | 23.8 | 73.3 | 82.7 | 49.3 | 83.6 | 114.0 | 66.9 | 91.3 | 75.0 | 73.9 | 91.2 | 51.9 | 88.1 | 68.4 | 37.2 | 55.8 | 50.5 | 64.9 | 51.3 | 93.7 |
| miscellaneous | % of use | 18.4 | 23.0 | 9.0 | 16.2 | 24.3 | 9.1 | 25.0 | 11.1 | 41.8 | 19.0 | 12.7 | 27.0 | 30.4 | 14.9 | 21.2 | 12.0 | 16.0 | 25.5 | 22.4 | 20.0 | 16.8 | 22.8 | 22.6 | 14.3 | 10.8 | 22.3 | 28.1 | 14.6 |
| polymers | % of use | 64.0 | 29.1 | 33.2 | 57.0 | 91.1 | 54.3 | 77.0 | 72.6 | 32.4 | 57.2 | 58.4 | 79.9 | 30.1 | 38.6 | 60.1 | 77.3 | 72.5 | 25.9 | 45.2 | 70.6 | 54.7 | 65.4 | 87.9 | 49.5 | 87.2 | 81.8 | 20.5 | 50.1 |
